# New Methods of Treatment in Phthisis

**Published:** 1891-10-10

**Authors:** 


### The Practitioner's Mirror and Retrospect.

[*The Editor will be glad to receive offers of co-operation and contributions from members of the profession. All letters should be addressed to* The Editor, The Lodge, Porchester Square, London, W.]

### MEDICINE.

### NEW METHODS OF TREATMENT IN PHTHISIS.

II.—The Gibbes-Shurly Method.

Well may we take a leaf out of a recent contemporary editorial which says: "When we read of the value of the inhalation of chlorine gas and the use of iodine and chloride of gold and sodium hypodermically in the treatment of pulmonary consumption," we cannot but think of the goats' blood system, the sulphuretted hydrogen system, the tuberculin and cantharidinate of potash systems, and all the rest; and we may be excused if we do not exhibit any eagerness to accept any further "systems." The originators of this line of treatment, however, both being men "above reproach," we cannot afford to dismiss any work of theirs without paying it at least the compliment of examining it.*

The method is based on certain theories as to the pathology of phthisis. Dr. Gibbes says that we have in phthisis two forms of consolidation in the lungs, the one brought about by inflammatory action from first to last, the other by a new growth; and the tubercle baccilli are always found in the first when caseation has taken place, but are absent altogether in a few cases of the second form. In the face of these facts, which can all be verified at any time, "where is the unity of phthisis?" asks the author, and how is it proved that the tubercle bacillus of Koch is the virus of either of these forms? According to him, in those processes where you find the tubercle bacilli, there is true tuberculosis, no matter what the maroscopical pathological picture is, or what the clinical evidence may show.

Now, what is the case as it stands at the present time? Consolidations are formed in the lungs, which break down and form cavities. These consolidations were considered by such authorities as Niemeyer and others to be formed in different ways, and to differ in their structure, and that this difference exists Dr. Gibbes is prepared to demonstrate. The discovery of the bacillus, however, upset this, and the unity of phthisis was taken as an accepted fact, and everything called tuberculosis when the tubercle bacillus can be found.

Dr. Gibbes summarises the views he holds on the pathology of the disease as follows: Firstly, he is convinced that we must recognise two forms of disease which result in the formation in the lungs; and, secondly, we must admit that one of these—pulmonary phthisis—occurring after broncho-pneumonia, is a purely inflammatory disease from beginning to end; thirdly, we must recognise two distinct lesions in the lungs in acute miliary tuberculosis, so marked as to constitute two separate diseases; fourthly, after due consideration, he thinks that we should admit that it is highly improbable that the tubercle bacillus can in one case set up an acute inflammation, resulting in breaking down and destruction of tissue, and in another case in the same organ cause a new growth of fibrous connective tissue without any inflammation whatever. Dr. Gibbes quotes Koch himself, the discoverer of the "cause" of tuberculosis, as having put forth a so-called cure which professedly leaves the cause of the disease untouched, and only attacks the results of the disease. It is a well-known fact that in any consolidation in the lungs, however small, where caseation has taken place, a number of different micro-organisms are found. They are all sharply differentiated from Koch's tubercle bacillus by their reaction to staining agents, as tubercle bacilli are alone able to hold the red colour when subjected to the action of an acid. This peculiarity is in some way associated with caseation, and it seems certain that this reaction is closely related to certain fatty materials. If any ordinary bacteria be cultivated on solid media, and a thin layer of butter be placed on them, so that they are covered with it, these bacteria will show the same reaction as the tubercle bacilli; that is, they will hold the red stain when passed through acid. Dr. Gibbes has shown that the bacilli found in megma have precisely the same reaction, until they have been cultivated on gelatin for some little time, when they lose this staining peculiarity. We may, therefore, consider that the reaction characterising Koch's tubercle bacilli may be acquired from the fatty material in the caseation, where the bacillus grows so abundantly that it evidently finds suitable pabulum. Now if its presence in the consolidation of broncho-pneumonia which has gone on to pulmonary phthisis can be satisfactorily explained, it seems to the author that there is no possible reason for including this disease under the head of tuberculosis. The bacilli developed in such enormous number in a patch of caseating consolidation, simply set to work and devour the devitalized patch, bring it into a softened condition, in which it can be passed off by the air passages. Dr. Gibbes states that it is most important that we should realise the different stages of this disease, and how it differs from tuberculosis, as, if we have this knowledge, it is not a difficult matter to cure it if taken early enough.

Tuberculosis is a much more formidable disease. It commences insiduously, and is often far advanced before its presence can be detected. It has this pecularity, that after growing to a certain size, the centre undergoes a necrotic change and loses its vitality. Dr. Gibbes found that Dr. Shurly working quite independently, had come to the same views as to the differentiation we have so briefly quoted, and in regarding the bacillus as an accessory rather than the actual factor in the production of tuberculosis. They shared the same view, too, that there was some morbid product responsible for the irritation resulting in this new growth of true tubercle. Working together on monkeys, they endeavoured to discover some way of neutralising this morbid chemical irritant. After numberless trials, they found that they could do this with iodoform, but that substance set up a fatty change in the liver, and had to be discarded. They afterwards found that iodine would prevent the formation of tubercle in inoculated animals, and that the chloride of gold and sodium would render tubercle bacilli innocuous, without, however, killing them. Chlorine gas, too, was found to be one of the most efficacious of these substances. Regarding chlorine gas, there are two methods of administering it. First, the gas may be evolved from chlorinated lime by the addition of dilute hydrochloric acid. Before the gas is evolved the atmosphere should be well charged with a spray of saturated sodium chloride (about two ounces in a small compartment of 550 cubic feet). The patient should breathe through the nose and with the mouth closed, and the sittings should commence with two minutes, and be gradually increased to twenty to thirty minutes. One or two, and exceptionally three or four sittings, will be required daily. In laryngeal and mild cases, the chlorine water (U.S.P.) mixed with a saturated solution of salt ($\frac{1}{2}-\frac{3}{4}-\frac{1}{4}$) should be vapourised in from ℥ss.—℥ii. at a sitting. In laryngeal phthisis weaker solutions and more frequent sittings may be used with a face inhaler. These chlorine inhalations are said to prevent further caseations, and it is irrespirable unless diffused in vapour of chloride of sodium.

Hypodermic injections, too, may be made. The instru-

*Journal Laryng., August, 1891.

ment should be provided with either gold or platinum-plated points. The injections are made in the gluteal region, beginning with iodine, gr. $\frac{1}{12}$ daily, gradually increasing the dose until grain $\frac{1}{2}$—1 is attained, then the gold and sodium should be injected, beginning with grain $\frac{1}{30}$—$\frac{1}{20}$ daily, gradually increasing $\frac{1}{5}$—$\frac{1}{3}$ grain, and if the medication is to be continued this should be reduced to $\frac{1}{10}$ grain daily. It will be better to alternate the gold and iodine injections daily. Should albumen appear in the urine the iodine must be suspended, and the gold only used. The continuance of large doses of the gold and sodium salt may produce a golden colour of the skin, which may possibly be attributed to bile. At the commencement of the treatment, loss of weight, sweating, feverishness may occur. The expectoration soon becomes lessened and watery later on. Asthma and anorexia may supervene, diarrhœa and dryness of the throat, and langour and increased pulse-rate. These symptoms only occur in a small percentage. The injection of gold and sodium in these cases is followed by a tonic reaction. In large doses of the gold and sodium, vertigo and nausea may appear occasionally. Most patients do better on small doses. One constant feature in cases showing rapid improvement is the supervention of asthmatic symptoms.

This is briefly the outline of the treatment applied in twenty-seven cases of which detailed records are given.

The physical signs seem generally to have shown improvement, and the bacilli to have disappeared from the sputa, and the patients improved in general condition.

Take one of these for example, Miss J. C., with a well. marked family history of phthisis. She dated her illness from a chill, followed by cough, fever, night-sweats, and an attack of hœmoptysis. Was first seen on January 1st, 1890, she was in the following condition: Pulse, 96; frequent cough and little expectoration; temperature, 100°; respiration, 26; skin dry; tongue coated; no diarrhœa, but poor appetite; says she is easily tired, and has pain in left chest and shortness of breath upon exertion; menstruation scanty. Physical signs: Slight dulness on percussion over upper left front; bronchial respiration diminished, fremitus, and exaggerated vocal resonance, with dry crackling on forced respiration over same region extending down to third rib. Treatment consisted of counter-irritation, rest, and the administration of quinine and iodoform with creasote, and thymol inhalation. She gradually grew better, and in the middle of February resumed her duties of teaching at school, which she continued for part of each day. After continuing in much same condition till May, she became worse, and had slight hæmoptysis. Temperature ranged from 98° to 101°; more pain in chest, and more cough and expectoration; night sweats appeared, and diarrhœa for a few days. She was treated with quinine and salicin, and rest enjoined. Physical exploration of the chest showed mucous râles over upper left front; expectoration showed bacilli. She continued to get worse in spite of various methods of treatment. About September 10th the temperature increased, as also the cough, pain in the chest, and expectoration. She was growing weaker, was very much emaciated and having night sweats again; hoarse and having sore throat; sputum contained tubercle bacilli. Physical signs then showed mucous râles and moist crackling over upper left, and cavernous respiration with creaking at left apex; bronchophonous, also broncho vesicular respiration and coarse râles over upper right front and over left back. She had redness and swelling of the upper larynx with uniform swelling of the arytenoids, ary-epiglottic folds and ventricular bands, but epiglottis not swollen; no erosion could be seen anywhere. She was now given hypodermic injections of 1-12th grain iodine daily, and an inhalation of chlorine water and solution of chloride of sodium. The iodine was increased $\frac{1}{2}$ grain the third week, when the gold and sodium was substituted, owing to the increased mucous expectoration, diarrhœa, and skin eruptions coming on. After the fourth week she received injections (and a few times the solutions mixed) every other day, and so on; she now receives one injection every two weeks. The inhalation of chlorine water was kept up till February 5th, since which time she has had none. October 18th she seemed worse, and so continued for about a week, having an exacerbation of fever and chills, with increased cough and expectoration. Since February 15th the temperature has shown no elevation; pulse 80 to 84; appetite and digestion good. She resumed her duties as teacher after holiday vacation, and has lost no time since. She has taken little or no medicine since early in January, and the physical signs clearly show retrogression of the process. Her voice is slightly husky, but larynx examined shows only a thickening of the ventricular bands. There is no odynphagia.

This may be taken as an average example of Dr. Gibbes' cases. We have endeavoured to give an outline of his method, and of Dr. Gibbes' pathological views on which it is based as nearly in his own words as our space permits. For fuller information we would refer to his articles in the *Therapeutic Gazette* for April 15th, and subsequent numbers. We cannot but recognise that his observations are based on prolonged clinical investigations, and perhaps many disappointments in recently vaunted cures tends to make one unduly sceptical, and we await further reports on this, the Shurly-Gibbes method, before we can come to any definite opinions in favour or against the method.

